# Sociodemographic factors and choice of oral anticoagulant in patients with non-valvular atrial fibrillation in Sweden: a population-based cross-sectional study using data from national registers

**DOI:** 10.1186/s12872-019-1029-z

**Published:** 2019-02-26

**Authors:** Venkatesh Kumar Gurusamy, Gunnar Brobert, Pareen Vora, Leif Friberg

**Affiliations:** 1 Bayer AG, Berlin, Germany; 2 Epidemiology, Bayer AB, Stockholm, Sweden; 3 Epidemiology, Bayer AG, Berlin, Germany; 4 Department of Clinical Sciences at Danderyd Hospital, Karolinska Institute, Stockholm, Sweden

**Keywords:** Non-vitamin K antagonist oral anticoagulants, Sociodemographic factors, Sweden

## Abstract

**Background:**

The Swedish healthcare system aims to provide equal access to care to all residents yet evidence suggests that patients with low socioeconomic status are less likely to receive new drugs. Associations between sociodemographics and prescription of non-vitamin K antagonist oral anticoagulants (NOACs) as an alternative to warfarin in Sweden have not been investigated.

**Methods:**

We conducted a cross-sectional study using linked national registers in Sweden. The study population included oral anticoagulant naïve patients aged ≥18 years with non-valvular atrial fibrillation (NVAF) who filled a first prescription for a NOAC or warfarin from 01 December 2011 to 31 December 2014. Multivariable logistic regression was used to identify factors associated with the choice of anticoagulant treatment; adjusted odds ratios (ORs) with 95% confidence intervals (CIs) were calculated.

**Results:**

Among 68,056 patients with NVAF, 27.4% (*N* = 18,638) started treatment with a NOAC and 72.6% (*N* = 49,418) started on warfarin. Patients starting treatment with a NOAC were more likely to be highly educated (OR 1.37, 95% CI: 1.30–1.45), in the highest income quartile (OR 1.23, 95% CI: 1.16–1.31) and have a leading professional occupation (OR 1.41, 95% CI: 1.27–1.58). Patients residing in rural areas were half as likely to start treatment with a NOAC as those in urban areas (OR 0.48, 95% CI: 0.45–0.51).

**Conclusion:**

Among Swedish patients with NVAF, those with high socioeconomic status and urban residence were more likely to start preventative treatment with a NOAC than warfarin. Future research should explore reasons for these inequalities in NOAC treatment.

**Electronic supplementary material:**

The online version of this article (10.1186/s12872-019-1029-z) contains supplementary material, which is available to authorized users.

## Introduction

Sweden has about 10 million inhabitants with more than 85% of its population living in urban areas. [1] The Swedish healthcare system is well regarded for delivering high quality and economically effective healthcare and is committed to ensuring equality in healthcare services irrespective of socioeconomic status, including equity in drug treatment. [2–4] Sweden has both public and private healthcare facilities that are largely publicly funded – approximately 80% of healthcare expenditure is tax funded. [2] The Swedish government regulates healthcare costs for both outpatient visits and medication prices, and key healthcare policies at both the national and county level are aimed at improving access to diagnosis and treatment. [2] In 2002, a reform to support the generic substitution of drugs was introduced where patients pay the difference in price between the prescribed drug and the generic substitution. [2] Since 2006, reforms have been implemented that aim to increase freedom for patients to choose care providers and treatment. [2] However, evaluations of Swedish healthcare system since such reforms have indicated socioeconomic inequalities in patient management and health outcomes. [5–8]

Since 2011, non-vitamin K antagonist oral anticoagulants (NOACs) – dabigatran, rivaroxaban and apixaban – have been available in Sweden as alternatives to warfarin for stroke prevention in atrial fibrillation (AF) and other indications. Although more expensive, this new class of drugs have more favourable benefit–risk profiles than warfarin [9–11] and can be prescribed in fixed doses without routine monitoring of coagulation. [12] National prescribing data for the period 2011–2017 show that NOAC use increased markedly during this period while warfarin use appeared to reach a plateau and started to decrease by the end of this period, suggesting that NOACs are becoming a preferred treatment over warfarin. [13] We used data from linked national registers for the period 2011 to 2014 to investigate associations between sociodemographic factors and initial treatment with a NOAC (vs. warfarin, the previous standard of care) in oral anticoagulant naïve patients with non-valvular atrial fibrillation (NVAF). To our knowledge, this relationship has not previously been investigated in Sweden, and findings may be relevant for other currently available new drugs compared with the standard of care.

## Methods

### Data sources

We used data from three national population-based registers in Sweden – the National Swedish Patient register, the National Swedish Dispensed Drug register, and the Longitudinal Integration Database for Health Insurance and Labour Market studies (LISA) register. The Swedish Patient register provides complete national coverage of hospital admissions since 1987. It also contains information about all visits to hospitals and hospital-affiliated outpatient clinics across Sweden since 2001. Variables in the register include date of admission and discharge, principal and secondary diagnoses and codes for surgical and other interventions. Diagnoses are coded according to the tenth revision of the International Classification of Diseases (ICD–10). The Swedish Dispensed Drug register contains details about every prescription dispensed from all pharmacies in Sweden since 01 July 2005. Completeness of data in the register is very high because all pharmacies in the country are required by law to participate, and information is automatically recorded (electronically) whenever a drug is dispensed. Medications dispensed to patients in long-term and community care are included in the register, although those given during acute hospitalizations are not. The LISA register includes detailed information of the entire Swedish population aged ≥16 years about educational level, occupation (including periods of unemployment/retirement), income and area of residence. Patient data from these registers can be linked through the use of unique 10-digit civic registration numbers, which are given to all residents in Sweden, and which are used in all patient contacts with the healthcare system and in contacts with authorities. The study protocol was approved by the local ethical review board (EPN 2014/876–31/4) and conforms to the Declaration of Helsinki.

### Study population and inclusion criteria

All patients aged ≥18 years with a diagnosis of NVAF in the National Swedish Patient register and a first prescription for a NOAC or warfarin in the Swedish Dispensed Drug Register between 01 December 2011 (when the first NOAC became available in Sweden) and 31 December 2014 were identified, with the date of first NOAC/warfarin purchase designated the index date. Patients who had previously received any oral anticoagulant from 1 July 2005 up to the index date were excluded. The study cohort therefore comprised patients naïve to anticoagulants prior to inclusion in the study and their first prescription for NOAC/warfarin identified from the register was their first ever treatment with an anticoagulant. We also excluded patients with a diagnosis of mitral stenosis and those with mechanical heart valve prosthesis because these conditions constitute mandatory indications for warfarin only.

### Covariates

Information on geographical area, immigrant status, education level, occupation and disposable income was obtained from the LISA register on the index date or any time in the previous 12 month using the most recent information. Occupation was categorized using the standard grouping from Statistics Sweden – the system on which research studies in Sweden using information on employment should use. [14] Information on patient comorbidities and comedications (in the 12 months before the index date) (Additional file 1: Table S1) were obtained from the National Patient Register. This information was used to calculate individual risk scores for stroke in patients with atrial fibrillation according to the well-known CHA_2_DS_2_-VASc and HAS-BLED scoring systems.

### Statistical analysis

Patients were grouped according to the first anticoagulant (NOAC or warfarin) received as treatment following their NVAF diagnosis. Differences in socioeconomic and demographic factors between the two groups were presented as means and standard deviations for continuous variables and as frequency counts and percentages for categorical variables. In the primary analysis, multivariable logistic regression was used to compute odds ratios (ORs) with 95% confidence intervals (CIs) for the association between socioeconomic/demographic variables and initial anticoagulant received (NOAC, coded as 1; or warfarin, coded as 0) adjusting for potential confounders, including comorbidities and co-medications filled with prescriptions. Variables were retained in the final regression model if they were significantly associated (*p* < 0.05) with the choice of anticoagulant after adjustment for age and sex, omitting those directly associated with each other to avoid problems with collinearity. Patients with missing data on a sociodemographic variable where classed into a stratum ‘missing for that variable. Other factors that were investigated for their association with receiving a NOAC as first anticoagulant treatment were: duration of AF (newly diagnosed within 3 months before the index date or diagnosed more than 3 months before the index date), and medical history including concomitant disease and medication. The final model included the following variables: sex, age, region, educational level, type of employment, disposable income, number of years since first AF diagnosis, calendar year of inclusion in the study, previous hospitalization for bleeding, anaemia, myocardial infarction, heart failure, valvular disease (other than the exclusion criteria), pacemaker or implantable cardioverter defibrillator, diabetes, chronic kidney disease, chronic obstructive pulmonary disease, alcohol index, dementia, hospitalization for falls occurring more than once, and use of the following drugs < 6 months before the index date: beta blockers, digoxin, class 1 antiarrhythmic drugs and sotalol. All analyses were performed using Stata version 14.

## Results

The study population comprised of 68,056 patients with a diagnosis of NVAF and a first prescription for a NOAC or warfarin. Of these, 18,638 (27.4%) received a NOAC as initial anticoagulant treatment and 49,418 (72.6%) received warfarin.

Table 1 presents the frequency of socioeconomic and demographic variables according to first anticoagulant prescription (NOAC or warfarin) and associations between these variables and receiving a NOAC as first anticoagulant therapy. The mean age of the NOAC group was slightly higher than in the warfarin group (74.4 years vs. 73.7 years, *p* < 0.001); approximately 45% in both groups were females. Patients receiving a NOAC as first anticoagulant therapy were less likely to live in rural areas of Sweden than patients started on warfarin (10.2% vs. 19.0%), and were more likely to have post-secondary education (≥9 years; 25.7% vs. 20.2%), to be in employment (26.1% vs. 21.3%), have higher clerk/leading position jobs (4.6% vs. 2.8%), have qualified white-collar jobs (5.8% vs. 4.2%) and have a higher income (mean 232,000 SEK vs. 209,000 SEK). The proportion of patients with missing data for education and for occupation was very low (< 2 and < 1%, respectively).

**Table 1:** ORs (95% confidence intervals) for the associations between socioeconomic and demographic factors and first anticoagulant prescription among patients with NVAF

Patient characteristic	NOAC	Warfarin	Crude OR (95% CI)	Age- and sex-adjusted OR (95% CI)	Multivariable adjusted OR^a^ (95% CI)
	*N* = 18,638	*N* = 49,418			
Sex					
Men	10,144 (54.4)	27,198 (55.0)	1.0 (ref)	1.0 (ref)	1.0 (ref)
Women	8494 (45.6)	22,220 (45.0)	1.02 (0.99–1.06)	1.04 (1.01–1.08)	1.05 (1.01–1.10)
Age (years)					
Mean ± SD	74.4 ± 11.1	73.7 ± 10.7			
18–54	964 (5.2)	2431 (4.9)	0.98 (0.91–1.06)	0.99 (0.91–1.07)	0.88 (0.80–0.97)
55–64	2488 (13.4)	6151 (12.5)	1.00 (0.95–1.06)	1.00 (0.95–1.06)	0.95 (0.88–1.02)
65–74	6456 (34.6)	15,969 (32.3)	1.0 (ref)	1.0 (ref)	1.0 (ref)
75–84	5630 (30.2	17,143 (34.7)	0.81 (0.78–0.85)	0.81 (0.78–0.84)	0.85 (0.81–0.90)
≥85	3100 (16.6)	7724 (15.6)	0.99 (0.94–1.04)	0.98 (0.93–1.03)	0.91 (0.85–0.97)
Region^b^					
Urban	9181 (49.5)	23,608 (47.9)	1.0 (ref)	1.0 (ref)	1.0 (ref)
South	3741 (20.2)	9821 (19.9	0.98 (0.94–1.02)	0.98 (0.94–1.03)	1.05 (1.00–1.14)
Mid	3721 (20.1)	6455 (13.1)	1.48 (1.41–1.55)	1.48 (1.41–1.55)	1.67 (1.59–1.77)
North	1890 (10.2)	9365 (19)	0.52 (0.49–0.55)	0.52 (0.49–0.55)	0.48 (0.45–0.51)
Education (years)					
≤9	6615 (35.5)	19,898 (40.3)	1.0 (ref)	1.0 (ref)	1.0 (ref)
9–12	6911 (37.1)	18,788 (38)	1.10 (1.06–1.15)	1.10 (1.06–1.15)	1.09 (1.04–1.14)
≥13	4785 (25.7)	9968 (20.2)	1.44 (1.38–1.51)	1.44 (1.38–1.51)	1.37 (1.30–1.45)
Missing	327 (1.8)	764 (1.6)	1.29 (1.13–1.47)	1.28 (1.13–1.47)	1.20 (1.00–1.43)
Occupation					
Not working (retired/unemployed)	13,765 (73.9)	38,873 (78.7)	1.0 (ref)	1.0 (ref)	1.0 (ref)
Higher clerk/ leading position	850 (4.6)	1357 (2.8)	1.77 (1.62–1.93)	1.97 (1.80–2.17)	1.41 (1.27–1.58)
Qualified white collar	1072 (5.8)	2077 (4.2)	1.46 (1.35–1.57)	1.60 (1.48–1.74)	1.20 (1.09–1.32)
Other white collar	430 (2.3)	959 (1.9)	1.27 (1.13–1.42)	1.39 (1.24–1.57)	1.06 (0.92–1.26)
Self-employed excl. Farmers	648 (3.5)	1548 (3.1	1.18 (1.08–1.30)	1.28 (1.16–1.42)	1.16 (1.04–1.30)
Farmer	201 (1.1)	550 (1.1)	1.03 (0.88–1.21)	1.12 (0.95–1.32)	1.03 (0.86–1.24)
Foreman/technician	37 (0.2)	60 (0.1)	1.74 (1.16–2.62)	1.96 (1.30–2.96)	1.74 (1.10–2.75)
Skilled within trade, service or care	494 (2.7)	1289 (2.6)	1.08 (0.97–1.20)	1.18 (1.06–1.32)	0.97 (0.85–1.10)
Skilled blue collar worker	360 (1.9)	900 (1.8)	1.13 (1.00–1.28)	1.29 (1.13–1.47)	1.04 (0.90–1.21)
Other blue collar worker	635 (3.4)	1566 (3.2)	1.15 (1.04–1.26)	1.29 (1.17–1.43)	1.08 (0.96–1.21)
Missing	146 (0.8)	239 (0.5)	1.72 (1.40–2.12)	1.87 (1.52–2.30)	1.62 (1.04–2.51)
Income (SEK) ^c^					
Mean (1000s) ± SD	232 ± 505	209 ± 296			
Quartile 1	4275 (22.9)	12,557 (25.4)	1.0 (ref)	1.0 (ref)	1.0 (ref)
Quartile 2	4411 (23.7)	12,806 (25.9)	1.01 (0.96–1.06)	1.02 (0.97–1.07)	1.00 (0.95–1.06)
Quartile 3	4490 (24.1)	12,499 (25.3)	1.06 (1.01–1.11)	1.09 (1.04–1.15)	1.02 (0.96–1.08)
Quartile 4	5462 (29.3)	11,556 (23.4)	1.39 (1.32–1.46)	1.46 (1.38–1.53)	1.23 (1.16–1.31)
Immigrant status					
Swedish background	16,503 (88.5)	43,930 (88.9)	1.0 (ref)	–	–
Foreign background	2135 (11.5)	5488 (11.1)	1.04 (0.98–1.09)	–	–

Data are n (%) unless otherwise specified

^a^Adjusted for sex, age, region, educational level, type of employment, disposable income, number of years since first AF diagnosis, calendar year of inclusion in the study, previous hospitalization for bleeding, anaemia, myocardial infarction, heart failure, valvular disease (other than the exclusion criteria), pacemaker or ICD, diabetes, chronic kidney disease, chronic obstructive pulmonary disease, alcohol index, dementia, hospitalization for falls occurring more than once and use of the following drugs < 6 months before the index date: beta blockers, digoxin, class 1 antiarrhthmic drugs and sotalol

^b^Urban = Stockholm, Malmö and Göteborg; South = Götaland; Mid = Svealand; North = Norrland

^c^Quartile 1 = < 130,000 SEK; Quartile 2 = 130,000–165,000 SEK; Quartile 3 = 165,000–238,000 SEK; Quartile 4 = ≥238,000 SEK)

*CI* confidence interval, *NVAF* non-valvular atrial fibrillation, *OR* odds ratio, *SD* standard deviation, *SEK* Swedish Krona

In the multivariable analysis, after adjusting for potential confounders including comorbidities and comedications, patients in rural areas of Sweden were half as likely be started on a NOAC than patients in urban regions of Sweden, OR 0.48 (95% CI: 0.45–0.51). Having university or college level education (post-secondary) was associated with a 37% higher likelihood of starting treatment with a NOAC compared with having compulsory level education (≤9 years). A higher likelihood of receiving a NOAC as initial anticoagulant was also seen for patients employed in higher clerk/leading positions (OR 1.41 95% CI: 1.27–1.58), qualified white collar workers (OR 1.20, 95% CI: 1.09–1.32) and for foreman/technician jobs (OR 1.74, 95% CI: 1.10–2.75), when compared with retired/unemployed patients. High income was also associated with a higher likelihood of initial NOAC treatment, 23% higher likelihood for patients in the highest income quartile, OR (1.23, 95% CI: 1.16–1.31) versus the lowest income quartile. Sex was not associated with the likelihood to receive a NOAC (OR 1.01, 95% CI: 1.01–1.10). Patients in the youngest (18–54 years) and oldest age groups (≥75 years) were less likely to receive a NOAC than those aged 65–74 years.

Patients with a first-ever diagnosis of AF made within 3 months before the index date were significantly less likely to be started on a NOAC than warfarin compared with patients with AF of longer duration; OR 0.63, 95% CI: 0.60–0.66. As expected, a strong association between the year of first anticoagulant prescription and NOAC prescription was seen; compared with 2013, ORs were 0.50 (95% CI: 0.39–0.63) for 2011, 3.20 (95% CI: 3.01–3.40) for 2013 and 12.40 (95% CI: 11.70–13.14) for 2014. Associations between comorbidities/comedications and first anticoagulant prescription among patients with NVAF are shown in Additional file 1: Table S2.

## Discussion

The results of our large population-based study in Sweden indicate that geographical location and several sociodemographic factors are associated with receiving treatment the newer NOACs rather than warfarin for stroke prevention. These are important findings in a country that aims to ensure equity to healthcare irrespective of sociodemographic background.

We found that patients living in rural areas were only half as likely to start treatment with a NOAC compared with those living in urban areas. The northern part of the country is sparsely populated and distances to hospitals, or even to general practitioners (GPs) could be very large – factors that might have been thought to favour treatment with a NOAC owing to the requirement for fewer blood coagulation monitoring visits. One possible reason for our opposite finding could be that fewer specialists and subspecialists exist in these remote areas. A registry study found that patients living in rural areas had a higher rate of GP appointments but a lower rate of hospital outpatient visits per 1000 inhabitants than those living in urban areas. [15] Another potential reason is because of local reimbursement regulations or recommendations within county councils, for example, some counties provide incentives to physicians to prescribe cheaper drugs as part of controlling costs. Finally, another reason might be that physicians would rather use warfarin to keep closer surveillance of their patients through more frequent visits to maintain adherence to treatment.

Higher education, higher income and professional occupations were all associated with an increased likelihood of starting preventative anticoagulant therapy with a NOAC, consistent with previous research on newly-marketed drugs. [16–20] Low educational attainment has previously been associated with a lower probability of using newly-marked drugs in general among the elderly Swedish population, irrespective of age, sex, comorbidity and type of residential area. [16] In the US, a national study of 4670 patients with recent diagnosed atrial fibrillation (75% of whom were started on a NOAC and 25% of whom were started on warfarin) also found higher patient education level to be associated with NOAC selection (vs. warfarin) after adjusting for confounders. [17] Another study, conducted in the Czech general population, found that at the district level, educated inhabitants were more likely to use new cardiovascular drugs. [18] Evidence from studies conducted among residents of Ontario, Canada suggest that higher neighborhood median income level is associated with a small increased likelihood of receiving newer antipsychotics, ocular beta-blockers^14^ and dabigatran (compared with warfarin). More professional type employment and income are intrinsically linked – and explanations for the small associations seen between them and NOAC prescription in this study are plausible. Highly-educated individuals may be better informed about new medicines and may be more likely to request these from prescribers [21] being able to better articulate their needs and preferences. They may be more likely to have private health insurance through their employer, leading to quicker access to specialty care. Additionally, there is evidence that prescribing specialty, especially cardiology, is associated with NOAC prescription in treatment naïve patients. [17, 22] Added to this is that, although medication in Sweden is subsidized, the ceiling for maximum expenditure for pharmaceuticals within a 12-month period could mean that lower-income patients may be less inclined to use newer, more expensive anticoagulants.

While the finding that elderly patients were less likely to receive a NOAC than those aged 65–74 years is supported by findings from other studies, [22] further data supporting our finding of those younger than 65 years were also less likely to receive a NOAC are lacking. We found that women were just as likely as men to be prescribed a NOAC, in contrast to findings from a previous study, which reported that among individuals with low educational attainment, use of newly-marketed drugs were less likely to be prescribed to women than men. [16] However, comparison is limited because stratification by both gender and educational level was not performed in this present study.

The main strengths of this study are the large study population and its representativeness of the Swedish population, thus the results of this study can be considered generalizable to the Swedish adult NVAF population as a whole. Although indications for treatments prescribed to patients who are managed exclusively in primary care may not be identified from the Patient Register, most patients with NVAF are likely to have been included – it is likely that only a small proportion of patients would not have had any hospital contacts at all during the lengthy observation period. A limitation of our study is that, while medications dispensed to patients in the long-term and in community care are included in the register, those given during acute hospitalizations are not. The register also does not include information on over-the-counter drugs. Adjustments for confounding variables were made in the regression analysis, yet residual confounding is possible; for example, data were not available for smoking and other lifestyle factors. There may also have been some misclassification in the CHA2DS2-VASc risk score due to underreporting of component variables. We expect this to be non-differential misclassification and would likely not substantially affect the main results.

## Conclusions

In conclusion, our study suggests the existence of socioeconomic and demographic inequalities in use of NOACs for stroke prevention in Swedish patients with NVAF. Further research is needed to explore reasons for such inequalities in NOAC treatment in a health system that aims to ensure equity to all citizens. Lines of investigation should focus on the role of patient groups, providers and other healthcare system factors in the prescription of anticoagulants in Sweden. Physicians’ early adoption of new drugs, preferential prescribing, susceptibility to patient demands and physician specialty in prescribing anticoagulants, all need to be investigated. Examination of country-specific treatment guidelines and incentive programs, and patient preferences are also worthy lines of investigation.

## Additional file

Additional file 1: **Table S1.** ICD-10 codes used to define comorbidities. **Table S2**. ORs (95% confidence intervals) for the associations between comorbidities/comedication and first anticoagulant prescription among patients with NVAF. (DOCX 52 kb)

## Abbreviations

AF: Atrial fibrillation
CI: Confidence interval
GPs: General practitioners
ICD: International Classification of Diseases
LISA: Longitudinal Integration Database for Health Insurance and Labour Market studies
NOACs: Non-vitamin K antagonist oral anticoagulants
NVAF: Non-valvular atrial fibrillation
OR: Odds ratio
