# Supplementary material for: Sociodemographic factors and choice of oral anticoagulant in patients with non-valvular atrial fibrillation in Sweden: a population-based cross-sectional study using data from national registers
Source: BMC Cardiovasc Disord. 2019 Feb 26;19:43. doi: 10.1186/s12872-019-1029-z (PMC6390327; doi:10.1186/s12872-019-1029-z)
Supplement: Supplementary file 1 — Table S1. ICD-10 codes used to define comorbidities. Table S2. ORs (95% confidence intervals) for the associations between comorbidities/comedication and first anticoagulant prescription among patients with NVAF. (DOCX 52 kb) [file 12872_2019_1029_MOESM1_ESM.docx]

**Supplementary Table 1.** ICD-10 codes used to define comorbidities.

| **Condition** | **ICD–10 or procedure code beginning with** |
| --- | --- |
| Ischaemic stroke or systemic emboli | I63–64, I693–694, I74 |
| Transient ischaemic attack | G45 |
| Intracranial haemorrhage | I60–62, S064–066 , I690–692 |
| Major bleeding | Hospitalization with any of the following diagnoses I60–62, S064–066 , I690–692, I850, I983, K226, K250, K252, K254, K256, K260, K262, K264, K266, K270, K272, K274, K276, K280, K284, K286, K290, K625, K661, K920, K921, K922, N02, R319, N95, H431, R04, R58, D629, procedure code DR029 |
| Anaemia | D50–64 |
| Myocardial infarction | I21–22 |
| Vascular disease | I21, I22, I252, I70–73 (as in CHA2DS2–VASc) |
| Heart failure | I50,I110,I130,I132,I255,K761,I42–43 |
| Valvular atrial fibrillation | I342, I050, I052, Q232, Z952 (mitral stenosis or mechanical heart valve) |
| Other valvular disease | I34–39, I05–08, Q22–23 except valvular atrial fibrillation |
| Pacemaker or implantable cardioverter defibrillator | Z950, Z450, procedure code FPE |
| Hypertension | I10–15 |
| Diabetes | E10–14 or use of antidiabetic drug (ATC codes beginning with A10) |
| Chronic kidney disease | N18–19, procedure codes DR016, DR024, KAS00, KAS10, KAS20 |
| Liver disease | K70–77, procedure codes JJB, JJC |
| Chronic obstructive pulmonary disease | J43–44 |
| Cancer within 3 years | Chapter C except C44 (basalioma) within preceding 3 years |
| Alcohol index^*^ | E244, F10, G312, G621, G721, I426, K292, K70, K860, O354, P043, Q860, T51, Y90–91, Z502, Z714 |
| Dementia | F00–03, F051, G300–301, G308–309 |
| Frequent faller | ≥2 hospitalizations with diagnosis W00–19 or R296 |
| CHA_2_DS_2_–VASc score | 1 point each for : heart failure, hypertension, age 65–74 years, diabetes, vascular disease, female sex and 2 points each for age ≥75 years and thromboembolism |
| HASBLED score | 1 point each for hypertension, renal failure, liver disease, thromboembolism, any bleeding, age ≥65 years, prescription of antiplatelet agent or NSAID, alcohol index |

^*^Codes used by the Swedish Board of Health and Welfare for annual reporting alcohol-related mortality in the population.

ICD, International Classification of Diseases

**Supplementary Table 2**. ORs (95% confidence intervals) for the associations between comorbidities/comedication and first anticoagulant prescription among patients with NVAF.

| **Patient characteristic** | **NOAC**  **N=18,638** | **Warfarin**  **N=49,418** | **Crude OR  (95% CI)** | **Age- and  sex-adjusted OR  (95% CI)** | **Multivariable adjusted OR^*^  (95% CI)** |
| --- | --- | --- | --- | --- | --- |
| **Comorbidities** |  |  |  |  |  |
| CHA2DS2-VASc score  (mean ± SD) | 3.24 ± 1.82 | 3.38 ± 1.79 | – | – | – |
| 0–1 point | 3271 (17.6) | 7358 (14.9) | 1.0 (ref) | 1.0 (ref) | 1.0 (ref) |
| 2–3 points | 7604 (40.8) | 19,303 (39.1) | 0.89 (0.84–0.93) | 0.81 (0.77–0.86) | 0.94 (0.89–1.00) |
| ≥4 points | 7763 (41.7) | 22,757 (46.1) | 0.44 (0.43–0.46) | 0.66 (0.62–0.71) | 0.81 (0.76–0.87) |
| Previous ischaemic stroke/systemic embolism | 2661 (14.3) | 6,870 (13.9) | 1.03 (0.98–1.08) | 1.05 (1.00–1.10) | – |
| Previous TIA | 1135 (6.1) | 2993 (6.1) | 1.01 (0.94–1.08) | 1.02 (0.95–1.10) | – |
| Previous intracranial haemorrhage | 301 (1.6) | 581 (1.2) | 1.38 (1.20–1.59) | 1.40 (1.22–1.61) | 1.29 (1.10–1.52) |
| Previous major bleeding | 1906 (10.3) | 4753 (9.6) | 1.07 (1.01–1.13) | 1.09 (1.03–1.15) | 1.10 (1.03–1.18) |
| Anaemia | 1637 (8.8) | 4630 (9.4) | 0.93 (0.88–0.99) | 0.94 (0.89–1.00) | 0.94 (0.87–1.00) |
| Previous myocardial infarction | 2012 (10.8) | 7138 (14.4) | 0.72 (0.68–0.76) | 0.72 (0.69–0.76) | 0.73 (0.69–0.76) |
| Heart failure | 3627 (19.5) | 11,315 (22.9) | 0.81 (0.78–0.85) | 0.82 (0.79–0.86) | 0.88 (0.83–0.92) |
| Valvular disease  (other than exclusion criteria) | 1421 (7.6) | 5094 (10.3) | 0.72 (0.66–0.76) | 0.72 (0.68–0.77) | 0.76 (0.71–0.81) |
| Pacemaker/implantable cardioverter defibrillator | 1529 (8.2) | 3526 (7.1) | 1.16 (1.09–1.24) | 1.18 (1.11–1.26) | 1.29 (1.20–1.38) |
| Hypertension | 11,393 (61.1) | 30,496 (61.7) | 0.98 (0.94–1.01) | 0.99 (0.95–1.02) | – |
| Diabetes mellitus | 3007 (16.1) | 9246 (18.7) | 0.84 (0.80–0.87) | 0.84 (0.80–0.88) | 0.88 (0.84–0.93) |
| Chronic kidney disease | 456 (2.5) | 2312 (4.7) | 0.51 (0.46–0.57) | 0.52 (0.47–0.57) | 0.47 (0.42–0.53) |
| Liver disease | 263 (1.4) | 603 (1.2) | 1.16 (1.00–1.34) | 1.15 (0.99–1.33) |  |
| Chronic obstructive pulmonary disease | 1265 (6.8) | 3737 (7.6) | 0.89 (0.83–0.95) | 0.90 (0.84–0.96) | 0.94 (0.87–1.01) |
| Cancer within previous 3 years | 1594 (8.6) | 4449 (9.0) | 0.95 (0.89–1.00) | 0.96 (0.90–1.02) | – |
| Alcohol index^‡^ | 616 (3.3) | 1407 (2.9) | 1.17 (1.06–1.28) | 1.16 (1.06–1.28) | 1.10 (0.99–1.23) |
| Dementia | 352 (1.9) | 679 (1.4) | 1.38 (1.21–1.57) | 1.42 (1.25–1.62) | 1.12 (0.97–1.29) |
| Frequent falls | 901 (4.8) | 1971 (4.0) | 1.22 (1.13–1.33) | 1.25 (1.15–1.36) | 1.10 (1.00–1.20) |
| **Medications** (Purchased within 12 months prior to index date) |  |  |  |  |  |
| Low-dose aspirin | 9058 (48.6) | 24,169 (48.9) | 0.99 (0.96–1.02) | 1.00 (0.96–1.04) | – |
| Clopidogrel | 1053 (5.7) | 2747 (5.6) | 1.02 (0.95–1.09) | 1.03 (0.96–1.11) | – |
| NSAID^§^ | 2288 (12.3) | 5957 (12.1) | 1.02 (0.97–1.07) | 1.01 (0.96–1.07) | – |
| Proton pump inhibitor | 4099 (22.0) | 10,626 (21.5) | 1.03 (0.99-1.07) | 1.03 (0.99–1.08) | – |
| Beta-blocker | 10,999 (59.0) | 27,919 (56.5) | 1.11 (1.07–1.15) | 1.11 (1.08–1.15) | 1.19 (1.14–1.24) |
| Verapamil/diltiazem | 359 (1.9) | 857 (1.7) | 1.11 (0.98–1.26) | 1.12 (0.99–1.26) | – |
| Digoxin | 1015 (5.5) | 1989 (4.0) | 1.37 (1.27–1.48) | 1.40 (1.29–-1.51) | 1.52 (1.39–1.67) |
| Class 1 antiarrhythmics | 486 (2.6) | 821 (1.7) | 1.58 (1.41–1.78) | 1.55 (1.38–1.74) | 1.22 (1.07–1.40) |
| Amiodarone | 115 (0.6) | 259 (0.5) | 1.18 (0.95–1.47) | 1.17 (0.94–1.46) | – |
| Dronedarone | 141 (0.8) | 331 (0.7) | 1.13 (0.93-1.38) | 1.09 (0.90–1.33) | – |
| Sotalol | 427 (2.3) | 964 (2.0) | 1.18 (1.05–1.32) | 1.17 (1.05–1.32) | 1.15 (1.01–1.32) |

Data are n (%) unless otherwise specified.

Note: Blank cells indicate relate to when those variables were not entered into the multivariable regression model because they were not significantly (at p<0.05) associated with the outcome after adjusting for age and sex, thus no multivariable adjusted OR was produced.

*Adjusted for sex, age, region, educational level, type of employment, disposable income, number of years since first AF diagnosis, calendar year of inclusion in the study,  previous hospitalization for bleeding, anaemia, myocardial infarction, heart failure, valvular disease (other than the exclusion criteria), pacemaker or ICD,  diabetes, chronic kidney disease, chronic obstructive pulmonary disease, alcohol index, dementia, hospitalization for falls occurring more than once and use of the following drugs <6 months before the index date: beta blockers, digoxin, class 1 antiarrhthmic drugs and sotalol.

^†^Fatal, intracranial or bleed requiring hospitalization

^‡^Using a set of codes used by the Swedish Board of Health and Welfare for annual reporting alcohol related mortality in the population.

^§^Over-the-counter purchases not included.

CI, confidence interval; NVAF, non-valvular atrial fibrillation; NSAID, non-steroidal anti-inflammatory drug; OR, odds ratio; TIA, transient ischaemic attack
